# Not Your Usual Case of Culture-Negative Endocarditis: A Case Report of Bartonella Endocarditis

**DOI:** 10.7759/cureus.24947

**Published:** 2022-05-12

**Authors:** Ricardo J Villasmil, John Sia, Ian Motie, Lisette Rodriguez, Natan Kraitman

**Affiliations:** 1 Internal Medicine, Sarasota Memorial Hospital, Florida State University College of Medicine, Sarasota, USA; 2 Infectious Disease, Sarasota Memorial Hospital, Florida State University College of Medicine, Sarasota, USA

**Keywords:** culture negative endocarditis, tetralogy of fallot, melody valve, bartonella, endocarditis

## Abstract

Advancements in transcatheter interventions have revolutionized the treatment of adult congenital heart disease. We present a case of a 32-year-old male with a history of tetralogy of Fallot with pulmonary atresia diagnosed with *Bartonella *spp. culture-negative infective endocarditis (IE) of his Melody valve, necessitating Melody valve replacement.

## Introduction

Infective endocarditis (IE) is an acute or subacute endocardial infection caused by bacterial, viral, and fungal organisms, and it is associated with a high rate of morbidity and mortality. The diagnosis is often challenging due to the wide range of clinical manifestations, but it should be considered in all patients presenting with fever and high-risk features including intravenous drug use, intracardiac devices, and prior valvular surgery. The diagnosis is based on clinical manifestations, blood cultures, and echocardiography. The diagnostic challenge is further complicated in cases where blood cultures remain negative, the incidence of which ranges from 2 to 7% of all cases [1]. Blood culture-negative endocarditis (BCNE) is defined as endocarditis without etiology following inoculation of at least three sets of intended blood cultures with negative cultures after seven days of incubation [2]. While generally rare, *Bartonella *spp. are among the most common causes of culture-negative endocarditis, posing a significant threat to immunocompromised patients and those with prosthetic heart valves. We report a case of a 32-year-old male presenting with *Bartonella henselae* culture-negative endocarditis of his Melody valve.

## Case presentation

A 32-year-old man presented with a one-month history of intermittent fevers as high as 103 ºF, chills, and dyspnea on exertion. He reported observing an intermittent bilateral upper extremity non-pruritic macular rash several weeks prior to the presentation. His past medical history included tetralogy of Fallot with pulmonary atresia requiring a Blalock-Thomas-Taussig shunt at birth. He had undergone complete repair including ventricular septal defect patch closure and a right ventricle (RV) to pulmonary artery homograft conduit at nine months of age. A cardiac catheterization and balloon dilation of the RV outflow tract (RVOT) with stent placement had been performed at age 11 with subsequent Melody valve placement at 25 years of age. He disclosed smoking tobacco and alcohol consumption socially but denied illicit drug use. He denied recent travel history, sick contacts, or animal exposure. Home medications included aspirin 81 mg, but he endorsed poor compliance. There was no known family history of congenital heart disease. On arrival, he was afebrile with a blood pressure of 101/71 mmHg, a heart rate of 99 beats/minute, and oxygen saturation of 99% on room air. Physical examination demonstrated a well-developed male in no acute distress. Cardiopulmonary examination revealed regular rate and rhythm with a normal S1, fixed split S2, and a grade III/VI systolic ejection murmur at the left upper sternal border. Skin examination demonstrated Janeway lesions in the right toe.

Investigations

**Figure 1:**
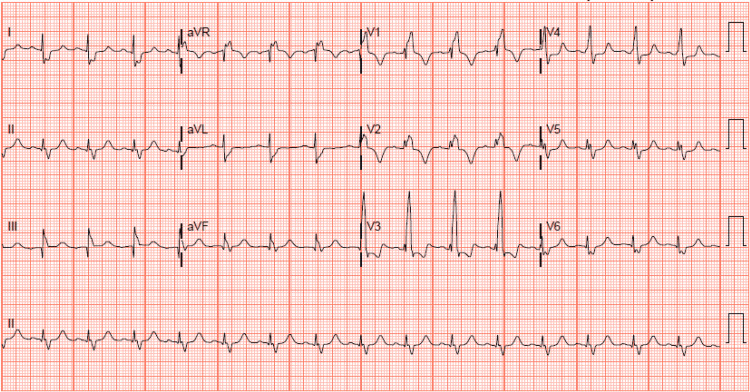
EKG showing sinus rhythm with right bundle branch block EKG: electrocardiogram

**Figure 2:**
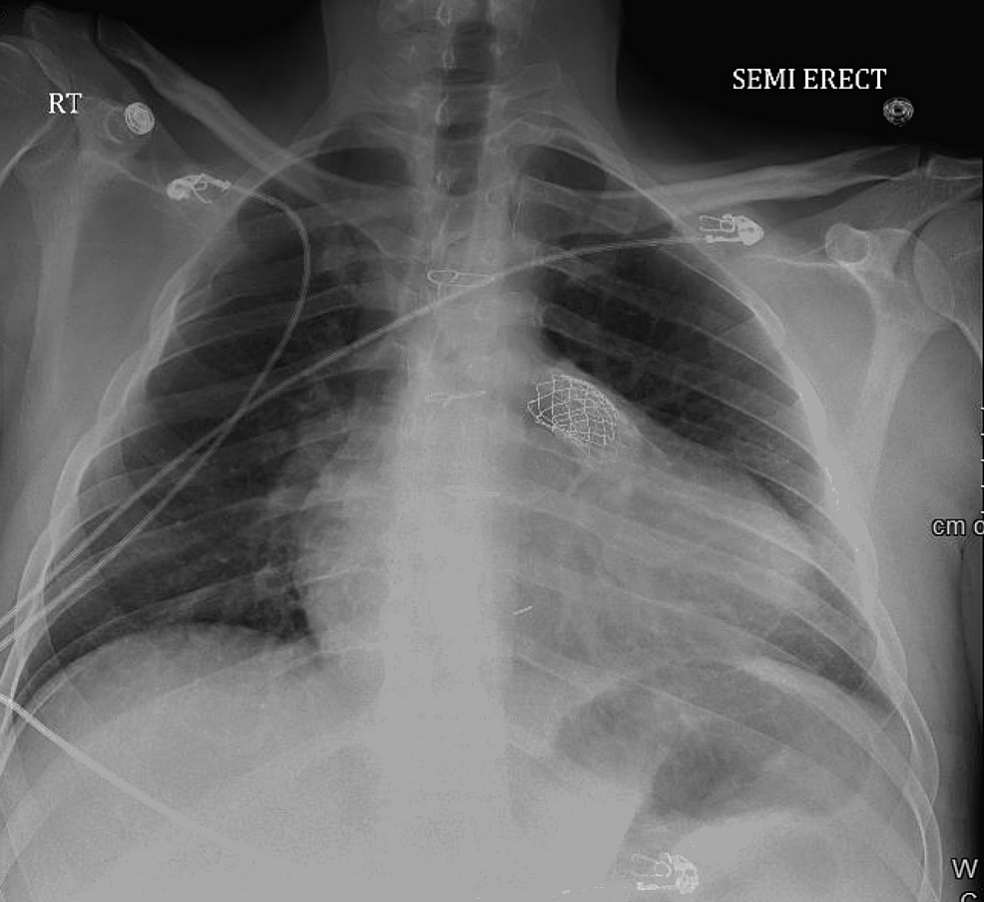
Chest radiograph demonstrating no acute cardiopulmonary process and stent in the RVOT RVOT: right ventricular outflow tract

**Figure 3:**
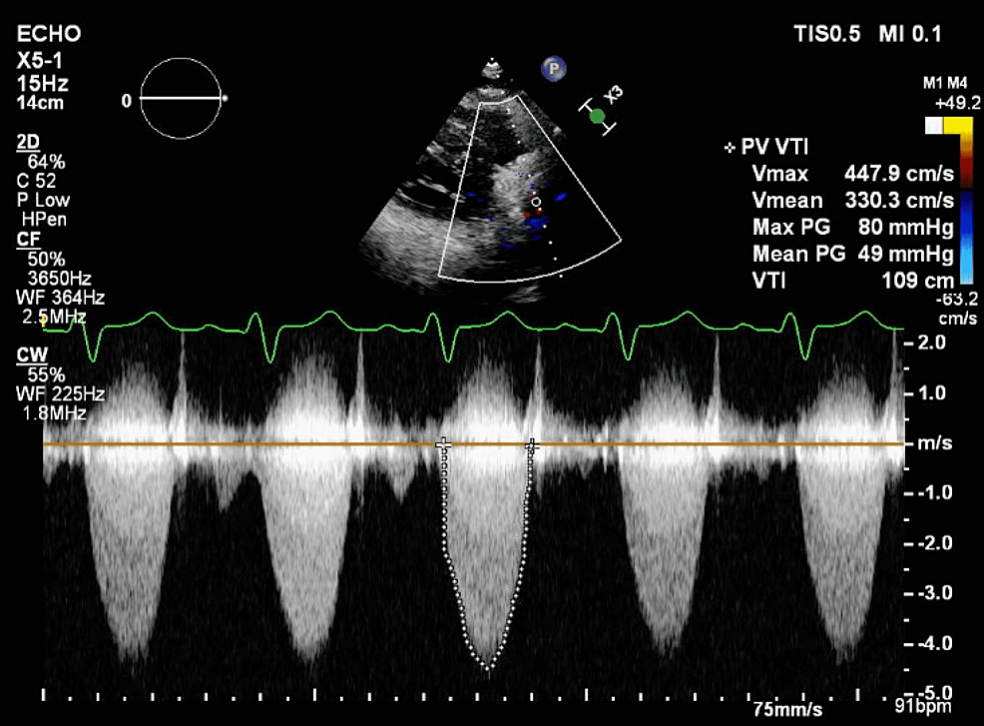
TTE showing significant severe obstruction across Melody valve TTE: transthoracic echocardiogram

A complete blood count showed pancytopenia. Blood chemistry showed creatinine of 2.17 mg/dl without a baseline value for comparison. The urinalysis showed a large amount of blood and protein. His proB-type natriuretic peptide (proBNP) level was 13,275 pg/mL. C-reactive protein and sedimentation rate were markedly elevated at 3.1 mg/dl and 71 mm/hr, respectively. EKG demonstrated sinus rhythm with a right bundle branch block (Figure 1). Serial troponins were negative. Chest X-ray displayed mild cardiomegaly with endograft overlying the left hilum (Figure 2). A transthoracic echocardiogram (TTE) revealed a normal ejection fraction, a small mobile mass in the proximal end of the RVOT with moderate to severe RVOT obstruction with a peak and mean gradient of 80 mmHg and 46 mmHg, respectively (Figure 3) (Video 1), which had increased significantly from the prior echocardiogram.

**Video 1:** TTE demonstrating significant abnormally thickened pulmonary valve TTE: transthoracic echocardiogram

Management

Blood cultures were obtained, and the patient was started on empiric antibiotics with vancomycin and ceftriaxone for suspected endocarditis. Despite empiric treatment with ceftriaxone and vancomycin, he continued to experience recurrent fevers. Due to concerns for Melody valve endocarditis, he was transferred to a tertiary center for adult congenital cardiology evaluation. Given his increased gradient and rising proBNP and decreasing RV function, he underwent cardiac catheterization with balloon angioplasty, stent placement, and placement of a new 22-mm Melody valve in the RVOT to relieve the obstruction. Blood cultures from both initial hospitalization and tertiary center remained negative. He remained afebrile and was discharged with plans to complete six weeks of outpatient antibiotics. He was subsequently hospitalized the following day for a fever of 102 ºF. Blood cultures were repeated, and antibiotics were broadened to cefepime and daptomycin. A repeat echocardiogram showed stable vegetation in the proximal aspect of the RVOT. Due to culture-negative endocarditis, further serology was obtained, which was positive for *Bartonella* and *Brucella*, with IgG titers of more than 1:2024 for *Bartonella henselae* and *Bartonella quintana*. He was IgM-reactive for *Brucella* with negative IgG. The antibiotic regimen was narrowed to doxycycline and rifampin to cover for both organisms, with subsequent defervescence. These antibiotics were dose-adjusted for renal function. The acute kidney injury was attributed to immune complex glomerulonephritis secondary to endocarditis, which improved following the antibiotic treatment. The patient was discharged to complete six weeks of antibiotic therapy with rifampin and doxycycline with consideration for chronic suppressive therapy. His symptoms improved following antibiotic therapy and the decision was made to provide chronic suppressive therapy with doxycycline with a repeat echocardiogram planned in six months.

## Discussion

Advancements in transcatheter interventions have transformed the management of valvulopathy in both adult and congenital heart disease. The Melody transcatheter pulmonary valve implantation (TPVI) was developed as an alternative to surgical reconstruction of the RVOT in patients with heterogeneous manifestations of congenital heart disease. The Melody valve has demonstrated satisfactory hemodynamic and clinical outcomes with freedom from surgical intervention at a rate of 91% in five years [3]. However, an important long-term complication is valvular dysfunction secondary to IE with the risk extending over several years following valve implantation with reported rates higher than those related to pulmonary homograft [4]. There is limited data exploring risk factors and outcomes of IE in TPVI, but a recent multicenter study has revealed that younger age, a prior episode of IE, and a higher residual gradient were associated with endocarditis [5]. The pathophysiologic mechanism driving endocarditis in this subgroup remains unclear; however, many theorize that thrombus formation in patients with poor compliance to aspirin therapy is an impactful first step [6]. Most cases involve common causative organisms of bacterial endocarditis; however, an underrecognized but important cause of BCNE such as *Bartonella henselae* has been only rarely reported but has demonstrated higher mortality rates and the need for surgical intervention [7].

Endocarditis caused by *Bartonella *spp.*,* a gram-negative coccobacillus, is a life-threatening infection that requires prompt diagnosis and treatment to prevent lethal complications. The variability in clinical presentations in patients without classic Oslerian manifestations makes the diagnosis often challenging. The current guidelines support the use of the modified Duke criteria to guide the diagnostic approach with specific serologic data dedicated to establishing pathogenic agents in culture-negative endocarditis [8]. Diagnosis made by serology and/or polymerase chain reaction (PCR) requires a *Bartonella* IgG titer >1:800 as a major criterion [9]. Establishing the diagnosis of prosthetic valve endocarditis (PVE) by utilizing the modified Duke criteria is challenging due to the poor sensitivity of blood cultures, and the lack of accurate imaging techniques to confirm valvular infection remains a barrier to clinical diagnosis. Current guidelines recommend using echocardiography, but it provides only modest sensitivity of 50% in identifying PVE and, unlike in left-sided endocarditis, TEE does not always improve diagnostic yield [7]. A systematic approach using multimodality imaging that employs echocardiography and novel imaging techniques such as PET/CTA can provide higher sensitivity ranging from 91-97% with better visualization, thereby improving diagnostic accuracy [10].

The treatment methods for *Bartonella* endocarditis are not clearly established in the literature. Many of the described empiric regimens include β-lactams and aminoglycosides, with 72% of patients requiring surgical intervention for the infected valve. Most guidelines recommend the utilization of doxycycline and gentamycin for a minimum of two weeks. However, in patients with renal insufficiency, rifampin should be utilized instead of gentamycin [11]. Further research is of vital importance to prevent this devastating complication and optimize its management.

## Conclusions

This case report highlighted the multifaceted diagnostic dilemma and the importance of a high index of clinical suspicion for *Bartonella *spp. species in blood culture-negative endocarditis in patients with Melody valve endocarditis. Innovations in transcatheter procedures have led to an increase in the number of patients who have undergone cardiac repair. This report described a strategic and multidisciplinary approach to identifying and treating this unique patient population.
